# Metastatic Fibrosarcomatous Transformation of Dermatofibrosarcoma Protuberans With Lung Metastasis: A Case Report

**DOI:** 10.7759/cureus.59742

**Published:** 2024-05-06

**Authors:** Salman Khan, Ekrem Yetiskul, Muhammad Niazi, Malik Waleed Zeb Khan, Ngowari Pokima, Georges Khattar, Terenig Terjanian

**Affiliations:** 1 Internal Medicine, Staten Island University Hospital, New York, USA; 2 Medicine, Khyber Medical University, Peshawar, PAK; 3 Hematology and Oncology, Staten Island University Hospital, New York, USA

**Keywords:** non-ossifying fibroma, skin lesions, dermatofibrosarcoma, sarcoma soft tissue, sarcoma

## Abstract

Dermatofibrosarcoma protuberans (DFSP) is a low- to intermediate-grade dermal soft tissue malignant tumor (sarcoma) with a high local recurrence rate but low metastatic potential. DFSP is characterized by uniform spindle cell fascicles arranged classically in a storiform pattern and by CD34 immunoreactivity. On gross examination, DFSP usually manifests as a white or yellow soft tissue mass with a smooth outer surface and poor circumscription. In this study, we report a case of DFSP with fibrosarcomatous transformation, a rare but well-known phenomenon encountered in DFSP that is correlated with an increased risk of adverse outcomes in patients with DFSP.

A 45-year-old male presented with a progressively enlarging lump on his left shoulder, initially suspected of being a lipoma but diagnosed as a fibrosarcomatous transformation of DFSP. Surgical resection was performed, with the subsequent identification of metastatic sarcoma in pulmonary nodules. Robotic-assisted thoracoscopy excised the nodules, confirming metastatic sarcoma with aggressive behavior. Despite negative adjuvant treatment plans, the patient remains under surveillance with imaging, showing no recurrence in recent scans. Continued follow-up with medical and surgical oncology is planned.

DFSP is a rare soft tissue sarcoma characterized by indolent growth and low metastatic potential, except in fibrosarcomatous transformation cases. Molecularly, DFSP is defined by a COL1A1-PDGFB fusion transcript that is targetable with imatinib therapy. Treatment involves wide surgical resection, with adjuvant radiation therapy in select cases. Radiation therapy may be employed in cases with close or positive margins, while conventional chemotherapy has limited utility. Multidisciplinary collaboration is crucial for optimal management.

Overall, this case underscores the challenges in diagnosing and managing aggressive sarcomas like fibrosarcomatous DFSP, emphasizing the importance of vigilant surveillance and multidisciplinary collaboration in optimizing patient outcomes. Further research is needed to understand the mechanisms underlying fibrosarcomatous transformation and to explore novel therapeutic avenues for this challenging malignancy.

## Introduction

Dermatofibrosarcoma protuberans (DFSP) is a low- to intermediate-grade dermal soft tissue malignant tumor (sarcoma) with a high local recurrence rate but low metastatic potential [1]. Approximately 80% to 90% of DFSP cases are low grade, whereas the rest of the cases are high grade due to the presence of a high-grade sarcomatous component (usually fibrosarcoma), which results in intermediate-grade sarcomas. DFSP usually manifests as a white or yellow soft tissue mass with a smooth outer surface and poor circumscription [1]. The standard diagnosis of suspected DFSP relies on a generous biopsy, i.e., a punch or excisional biopsy [1]. DFSPs are very less likely to metastasize (less than 5%); however, they have a propensity to recur locally. Repeated recurrence of the tumor is a risk factor for malignant transformation of sarcoma to DFSP-fibrosarcomatous (DFSP-FS). The lungs are reportedly the most common site of metastatic spread.

In this study, we report a case of DFSP with fibrosarcomatous transformation, a rare but well-known phenomenon encountered in DFSP that is correlated with an increased risk of adverse outcomes in patients with DFSP [2]. We also provide a detailed overview of the epidemiology of DFSP and DFSP-FS, as well as the distinct histopathological, immunohistochemical, and clinical features of DFSP-FS. Additionally, we discuss the association between p53 and MDM2 overexpression and the development of fibrotic sarcomatous variants of DFSP, and we also correlate the findings in the present case with the available literature.

## Case presentation

**Figure 1:**
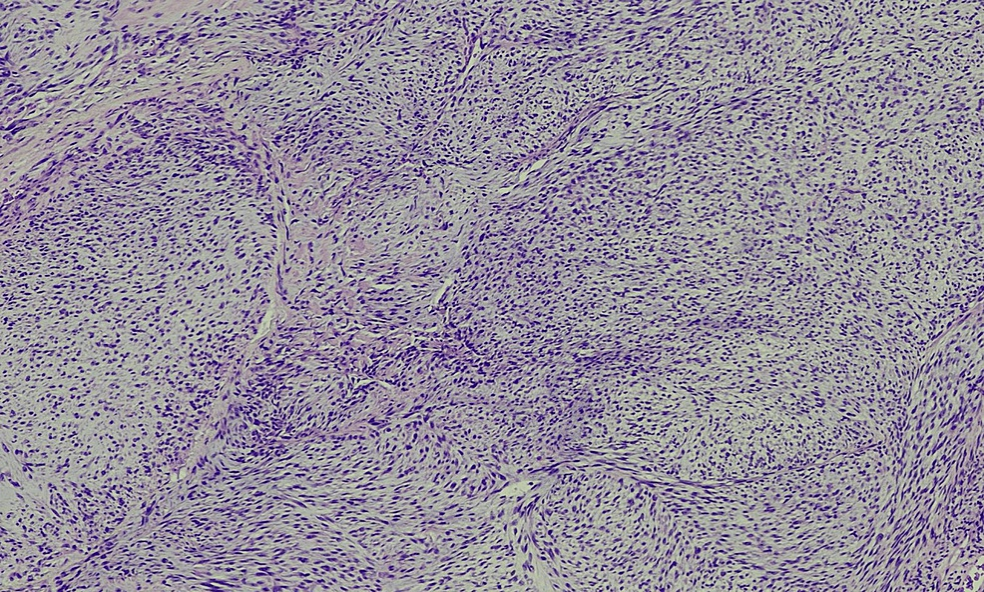
DFSP - low power: spindle cells with a nodular architecture growth pattern DFSP: dermatofibrosarcoma protuberans

**Figure 2:**
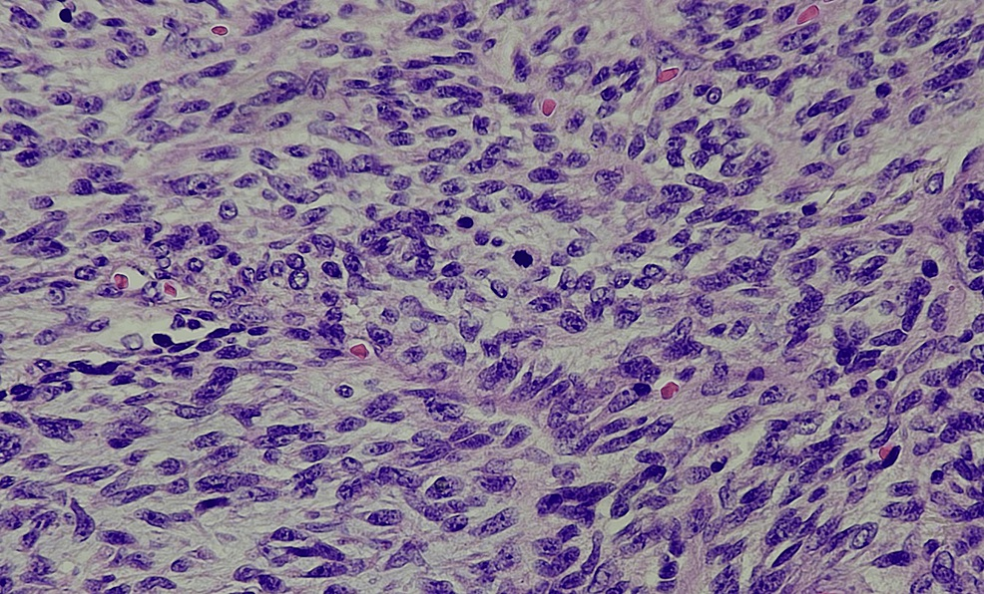
High power: high magnification demonstrating brisk mitosis, individual cells with prominent nucleoli, and hyperchromasia

Three years ago, a 46-year-old man noticed a small lump on his left shoulder area. With time, this mass grew and caused pain. An excisional biopsy was performed, which revealed an encapsulated, soft, and rubbery mass, hinting at a lipomatous tumor. The resected mass was 7 cm × 5.5 cm × 1.5 cm in size and tan-yellow in color. Pathology revealed a spindle cell neoplasm favoring sarcoma, extending into all the margins of the specimen. The differential diagnosis was liposarcoma, low-grade fibromyxoid sarcoma, or malignant peripheral nerve sheath tumor, among others. The specimen was observed to have spindle cells in cellular fascicles, arranged in intersecting fascicles in a herringbone pattern. The focal area showed lower cellularity, with plump spindle cells in a storiform pattern (Figure 1). Mitotic activity was increased in cellular areas that exhibited more than 20 mitotic figures in 10 high-power fields (HPFs) (Figure 2). Immunohistochemical staining was positive for CD34 and vimentin and negative for SMA, desmin, S100, CD117, CK AE1/AE3, and P53.

Overall, the findings were most consistent with the findings of fibrosarcomatous transformation of DFSP, which extends into the margins of the tumor. A PET scan was performed one month after resection and showed mild uptake over the left acromion surgical site and an indeterminate perivascular mediastinal density. The patient was arranged for wide local excision (WLE). During this time, the patient developed similar masses in other parts of the body. The patient underwent WLE of the primary tumor, and the new masses in the parascapular area and left upper arm were resected. The final diagnosis via pathology revealed hyalinized fibrosis in the parascapular and left upper arm masses with no evidence of neoplasm. The sample from the left shoulder mass was consistent with malignant spindle cell neoplasm and consistent with fibrosarcomatous transformation, measuring 3.5 cm in the greatest dimension. All margins of resection (including the peripheral skin margin and deep soft tissue margin) were free of neoplasms, without any evidence of lymphovascular invasion.

Approximately one year after the WLE of the DFSP, the patient presented with shortness of breath and cough, and a CT was performed that revealed new solid pulmonary nodules. A CT-guided biopsy showed no evidence of malignancy. However, on serial CT scans, these pulmonary nodules continued to grow over the course of the next year. Given that the previous fine needle aspiration was negative, the patient underwent robotic-assisted left thoracoscopy, left upper lobe apical posterior segmentectomy, and mediastinal lymph node dissection. Pathology confirmed that the mass was a malignant spindle cell neoplasm, which was consistent with metastatic sarcoma. All the mediastinal lymph nodes were negative for malignancy. Since the only site of recurrence registered by PET was in the lungs, and given the slow growth of the tumor, it was decided to excise the right lung nodule. The patient was then subjected to robotic-assisted right thoracoscopy and right upper lobe wedge resection without any complications. A PET scan was repeated two months after the surgery and revealed no evidence of pathologic FDG uptake.

Overall, the patient is doing well and is continuing to follow up with the medical oncology and surgical oncology teams. There is no plan for adjuvant treatment, and we will continue surveillance with imaging (CT and PET scans).

## Discussion

DFSP is an indolent dermal and subcutaneous soft tissue sarcoma of intermediate to low malignancy, except for the fibrosarcomatous variant (DFSP-FS). An epidemiological study [2] describing the incidence of DFSP in the United States revealed that the overall incidence was 4.1 per million in 10 years from 2000 to 2010, testifying to the rarity of this disease. The 10-year survival rate of DFSP patients is 99% [2]. DFSP commonly occurs in middle-aged adults [2]. The trunk is the most typical anatomical site, except in older men [2]. Older age, male sex, black race, and anatomic location of the limbs and head are associated with worse prognosis and survival [2]. DFSP has also been reported in people with immunodeficiency disorders such as X-linked agammaglobulinemia, adenosine deaminase-deficient severe combined immunodeficiency, ataxia telangiectasia syndrome, and HIV infection.

DFSP can initially manifest as a painless, slow-growing subcutaneous lesion or dermal plaque. Its presentation consists of sclerotic plaques that form from the confluence of small nodules or a keloid-like cutaneous plaque [1]. When left untreated, it can invade the local fascia, muscle, and bone and metastasize to other organs in later stages. Patients with DFSP are at increased risk of several subsequent primary malignancies, with much of the overall increased risk attributable to the increased risk of nonepithelial skin cancer. Under an electron microscope, the DFSP looks like stellate or spindle cells with long, slender, ramified cell processes joined by primitive junctions, similar to dermal dendrocytes [3].

The fibrosarcomatous transformation of DFSP is a well-known phenomenon and constitutes a high-grade DFSP. DFSP is characterized by the replacement of the characteristic storiform cellular arrangement of DFSP with long, gently sweeping fascicles of spindle cells that intersect at various angles, known as the herringbone pattern. A statistically significant difference existed between the mitotic rates of DFSP and DFSP-FS. Usually, the spindle cells of DFSPs strongly express CD34 and negatively express S-100, melan-A, alpha-smooth muscle actin, and factor XIIIa [1]. Notably, CD34 expression can be reduced in almost half of the fibrosarcomatous variants of DFSP [4]. Poor clinical outcomes were observed in patients with necrosis, a high mitotic rate, and pleomorphic areas in DFSP-FS.

Approximately 17% of DFSP-FS patients in the later stages (metastasis/recurrence) exhibit point mutations in the p53 gene, suggesting the involvement of p53 mutations and MSI in the progression of DFSP to fibrosarcoma, precisely as early and late events, respectively [2]. In a study comparing p53 overexpression in patients with DFSP and patients with DFSP-FS, three out of five patients with DFSP-FS had p53 overexpression without overexpression of MDM2 or p21, and the remaining two had p53 overexpression with MDM2 and p21 overexpression. Moreover, patients with DFSP with no fibrosarcomatous transformation had no p53 overexpression, MDM2, or p21. Patients with DFSP-FS and p53, MDM2, or p21 overexpression had frequent local recurrences with increasingly shorter intervals (mean 4.5 years). In contrast, only three out of the 13 patients with DFSP experienced local recurrence; these recurrences were limited by a longer recurrence interval (mean 10.3 years). This finding suggested the possible role of alterations in the p53 pathway in the development of fibrosarcomatous transformation in DFSP and suggested that p53 and MDM2 overexpression are correlated with the aggressive behavior of DFSP-FS as opposed to the milder behavior of ordinary DFSP and DFSP-FS without p53 alterations.

Genetically, DFSP involves the fusion of the COL1A1 (collagen type 1 alpha 1) and PDGFB (platelet-derived growth factor beta) genes, which occurs due to a translocation between chromosomes 17 and 22. This fusion results in the production of a protein called COL1A1-PDGFB. The PDGFB protein activates the PDGF receptor on tumor cells, leading to uncontrolled tumor growth. To treat metastatic DFSP, imatinib mesylate, a PDGFR inhibitor, has been used and shown to be effective. Imatinib is a small molecule that inhibits the tyrosine kinase pathway, thereby inhibiting cell growth and proliferation [5]. In 2006, imatinib was approved by the FDA for the treatment of DFSP, and in 2010, the NCCN included imatinib in its treatment guidelines [6]. The drug was not used in the current patient because the tumor was resected completely and there was no recurrence detected, nor was any residual disease seen. There was no need for any further treatment with medications.

Nevertheless, the definitive treatment for localized disease includes surgical resection [4] involving wide surgical margins, which includes WLE and Mohs micrographic surgery. DFSPs are radiosensitive tumors, but randomized clinical trials to support the use of radiation therapy for their treatment are lacking.

## Conclusions

DFSP is a rare soft tissue sarcoma characterized by indolent growth and low metastatic potential, except in fibrosarcomatous transformation cases. Molecularly, DFSP is defined by a COL1A1-PDGFB fusion transcript that is targetable with imatinib therapy. Treatment involves wide surgical resection, with adjuvant radiation therapy in select cases. Radiation therapy may be employed in cases with close or positive margins, while conventional chemotherapy has limited utility.

Overall, this case underscores the challenges in diagnosing and managing aggressive sarcomas like fibrosarcomatous DFSP, emphasizing the importance of vigilant surveillance and multidisciplinary collaboration in optimizing patient outcomes. Further research is needed to understand the mechanisms underlying fibrosarcomatous transformation and to explore novel therapeutic avenues for this challenging malignancy.
